# Molecular connections between inflammation and social determinants of health

**DOI:** 10.3389/fepid.2025.1683955

**Published:** 2025-11-11

**Authors:** Aditi Vijendra, Claire Kunkle, Jalin Jordan, Anna Erickson, Kingsley Osei-Karikari, Grace Ratley, Ian A. Myles

**Affiliations:** 1 Laboratory of Clinical Immunology and Microbiology (LCIM), Epithelial Therapeutics Unit, National Institute of Allergy and Infectious Diseases (NIAID), National Institutes of Health (NIH), Bethesda MD, United States; 2 Unit of Integrative Metabolomics, Institute of Environmental Medicine Karolinska Institute, Stockholm, Sweden

**Keywords:** social determinants of health, pollution, inflammation, environmental justice, disparities

## Abstract

Chronic inflammatory diseases such as autoimmune disorders, cancer, cardiovascular diseases and neurodegenerative disorders are a significant cause of morbidity and mortality in the industrialized world. Socioeconomically disadvantaged communities bear a disproportionately high burden of these inflammatory diseases. This review synthesizes evidence linking various domains of the Social Determinants of Health (SDoH)—economic stability, education access and quality, healthcare access and quality, neighborhood and built environment, and social and community context—to inflammatory pathways and mechanisms. Across domains, biological mechanisms such as cytokine dysregulation, toll-like receptor (TLR) activation, hypothalamic-pituitary-adrenal (HPA) axis alterations and gut microbiome disruption act together to sustain proinflammatory states that drive adverse health outcomes in marginalized communities. Although causality is obscured by interrelated determinants, identifying inflammation as a shared pathway between various determinants highlights the need for structural interventions to reduce chronic disease burden.

## Introduction

Incidence of Inflammatory diseases are rapidly rising and the prevalence of such diseases is anticipated to continue increasing in the coming decades (1). Chronic inflammatory diseases are recognized as the most significant causes of death in the industrialized world, as more than 50% of all deaths are attributed to diseases associated with inflammation (2). These conditions include allergies (3), metabolic disorders (4), cancer (5), autoimmune diseases (6), and neurodegenerative diseases (7). However, the burden of chronic inflammatory diseases is disproportionately bore by communities with higher rates of socioeconomic disadvantage and barriers to healthcare access (8). These broad social factors have been conceptualized by Healthy People 2,030 as social determinants of health (SDoH), which are organized into five domains: economic stability, education access and quality, health care access and quality, neighborhood and built environment, and social and community context (9). This review aims to highlight inflammatory exposures across the five SDoH domains and mechanisms by which social determinants have been proposed to influence chronic low-grade inflammation.

### Systemic inflammation and socioeconomic status overview

Inflammation is essential for fighting pathogens and malignant cells, alongside promoting tissue repair. However, systemic and chronic activation of the immune system underpins the development of inflammatory disease (10). Sub-clinical, systemic inflammation describes elevated expression of inflammatory molecules and heightened immune activity that may not yet manifest with overt clinical symptoms. The prolonged and systemic expression of inflammatory molecules is associated with harmful effects including oxidative stress, fibrosis, mitochondrial dysfunction, and cellular senescence (11). The sustained effects of inflammatory responses in the absence of a pathogenic target are largely associated with several mechanisms of chronic disease development. For instance, the prolonged production of reactive oxygen species by immune cells and corresponding oxidative stress are associated with further stimulation of an inflammatory response, creating damage associated with organ dysfunction and metabolic dysregulation.

In measuring systemic inflammation, Interleukin-6 (IL-6) and C-reactive protein (CRP) are frequently used biomarkers (12). IL-6 is synthesized at the beginning of many immune responses and has several effects ranging from promoting antibody production to inducing acute-phase protein synthesis (13). C-reactive protein is an acute-phase protein released by the liver in response to IL-6 production, and is commonly used as a non-specific inflammation marker (14). Furthermore, the systemic, heightened presence of leukocytes and expression of inflammatory cytokines in the absence of overt clinical symptoms are suggestive of chronic inflammation. Given the broad functions and triggers of inflammation, mechanisms and biomarkers of inflammation can vary vastly and have a wide range of effects (12). CRP and IL-6 have traditionally facilitated insights into the association between systemic inflammation and chronic disease but have been recently joined by many other measurements of inflammation (15). As an example, differential white blood cell counts, were shown to be predictive of all-cause mortality, alongside cancer, cardiovascular and cerebrovascular specific mortality (16).

**Figure 1:**
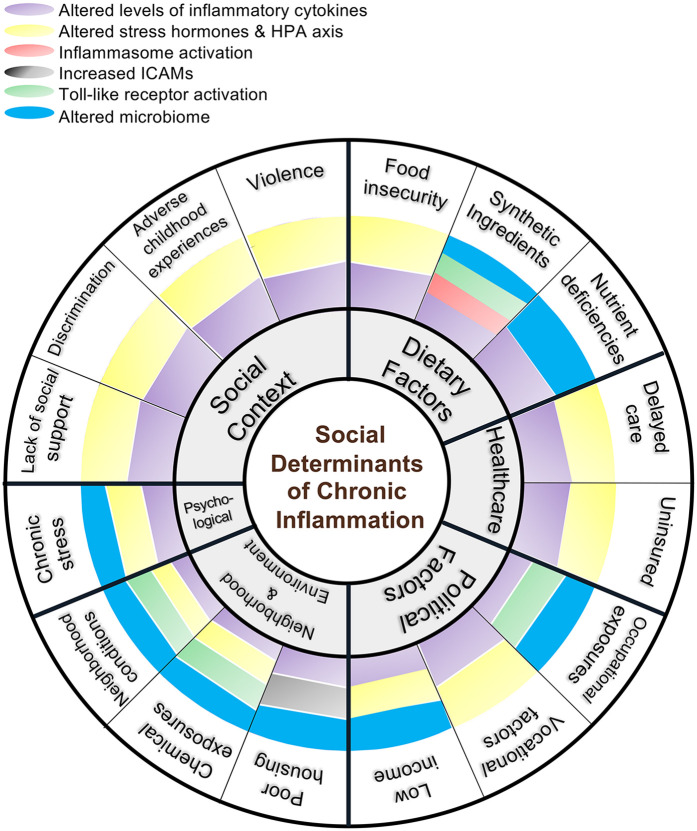
Integrative framework for social and biological determinants of chronic inflammation. This diagram maps social, environmental, and dietary conditions to specific immunologic pathways promoting inflammation and highlighting converging mechanisms—cytokine dysregulation, HPA axis alteration, innate immune receptor activation, and microbiome shifts.

A large body of evidence highlights the association of socioeconomic status (SES) with low-grade inflammation (summarized in Figure 1). For instance, a meta-analysis of 43 studies which assessed the relationship between socioeconomic status, during both childhood and adulthood, and biomarkers of systemic inflammation found a significant negative correlation between SES and CRP and IL-6 (17). Although the effect was attenuated after controlling for BMI and smoking, the correlation remained significant. Another systemic review investigating the relationship between childhood socioeconomic status and chronic inflammation revealed a significant association between parental finance and inflammation, as measured by CRP (18). Transcriptional profiling of placental biopsies and umbilical blood collected at birth suggested that both elevated immune activation and decreased fetal maturation are inversely associated with maternal deprivation (19). Maternal disadvantage was determined by income receipt of federal benefits, and education level. The significant association between SES and inflammation, reported across several studies, after adjustment for factors including BMI, suggests more research is necessary to further interrogate the relationship as to understand if and which causal mechanisms exist.

## Specific social determinants of health

### Dietary determinants

#### Food insecurity

Food insecurity and diet quality have been proposed to mediate the association between economic stability and low-grade inflammation (20). Low income is consistently associated with lower dietary quality scores, and socioeconomic disparities in diet quality have widened over time (21). A study examining diet quality among residents of disadvantaged neighborhoods found that a minority of residents met dietary guidelines (22). The low affordability of healthy food has been shown to explain substantial portions of the association between SES and diet quality (23, 24). However, beyond high costs of healthy food, researchers have also proposed biological mechanisms by which low SES further influences poor diet quality. The associated stressors and uncertainty of poverty, especially extreme poverty, have been shown to affect stress, appetite, and the hunger-related hormones which shaping eating habits (25). Furthermore, food insecurity has been suggested to promote dependence on energy dense, palatable diets and alter metabolism through stress-related mechanisms, including the hypothalamus-pituitary-adrenal (HPA) axis (26).

#### Nutrient deficiencies

Poor diet contributes to low-grade inflammation through several mechanisms, including the absence of necessary nutrients that are associated with healthy immune regulation. A study using National Health and Nutrition Examination Survey (NHANES) data compared nutrient and food group intake among children aged 2–5 years with the family income to poverty ratio (PIR). The results suggested children in the low PIR cohort had lower dietary fiber, dairy, and calcium intakes, alongside lower Healthy Eating Index (HEI) scores overall (27). Dietary fiber is associated with several processes that mitigate low-grade inflammation as it metabolized by intestinal microbial species into short chain fatty acids (SCFAs). The resulting SCFAs regulate the immune response through several mechanisms, including increased gene expression of NF-κB mediators (28), generation of regulatory T cells (29), and improving epithelial barrier integrity (30). Insufficient fiber intake may promote heightened inflammation by facilitating the translocation of damage-associated molecular patterns (DAMPs) due to compromised barrier integrity, as well as promote immune dysregulation by reducing SCFA signaling (31).

Alongside fiber, several notable anti-inflammatory micronutrients are less abundant in low-income diets. Anthocyanins and quercetin, for example, are flavonoids that are predominantly found in fresh foods. Flavonoids have been shown to decrease NF-κB expression (32) and attenuate expression of pro-inflammatory cytokines (33). However, flavonoid intake has been reported to be lower in low-income cohorts than in high-income counterparts (34). Additionally, vitamin D is a key nutrient associated with maintaining healthy immune function through its anti-inflammatory properties. In cell lines and peripheral blood mononuclear cells (PBMCs), treatment with vitamin D provided strong evidence of its anti-inflammatory properties, with marked decreases in anti-inflammatory cytokines including IL-6, TNF-α, MCP-1, and IL-10. Potential mechanisms of anti-inflammatory action by vitamin D included decreased protein expression of Toll-like receptors (TLR), lower levels of phosphorylated p38, and decreased reactive oxygen species (35). However, there is some evidence that vitamin D may be a reverse acute-phase reactant; meaning, that low levels of vitamin D may result from the presence of inflammation rather than be the cause of inflammation (36). However, there are some data from randomized controlled trials showing vitamin D supplementation resulted in improved regulation of inflammation, with reductions in CRP and TNF-α, alongside increased signaling of Regulatory T cells (37, 38). Like deficiencies in other key anti-inflammatory nutrients, vitamin D deficiency is positively associated with low income in the United States (39).

#### Intake of foods with synthetic ingredients

In addition to insufficient intake of key anti-inflammatory nutrients, intake of highly-processed and ultra-processed foods (UPF) is largely associated with increased inflammation and dysregulation of the immune system. Evidence suggests ultra-processed food consumption is on the rise, with increased availability in low-income communities (40). However, the definition of UPF however is controversial. Sugar, salt, and cooking oils—each of which have known inflammatory effects (41)—are not considered ultra-processed (42). Consuming a meal of bread, cheese, and tomato sauce would constitute consuming a processed diet but, combining them into a cheese pizza would be defined as an ultra-processed diet. While this manuscript cannot settle this debate, for purposes of simplicity we will consider UPF as those containing ingredients which are either synthetic or require industrial processing to extract because such foods are nearly always defined as ultra-processed. For example, adding hydrogenated oils or high-fructose corn syrup to an otherwise minimally processed recipe will beget an UPF designation.

Ultra-processed diets overwhelmingly lack previously described nutrients which maintain healthy functioning of the immune system (43). Alongside their low nutritional value, many UPF have artificially heightened levels of inflammatory ingredients, such as cholesterol and processed saturated fatty acids. While naturally occurring, saturated fats tend to include several different lipid moieties, whereas processed fats are typically restricted to the highly inflammatory palmitic acid and steric acid (41). Given that these specific saturated fats are components of bacterial lipopolysaccharide (LPS), they can activate anti-bacterial mechanisms through activation of TLR4 (44). Industrial processing and storage mechanisms necessary for UPF development have also been implicated in the formation of dietary cholesterol oxidation products, which are associated with several disease etiologies (45).

In *in-vivo* models, high cholesterol diets led to inflammasome activation in the intestinal epithelium, resulting in both local and systemic inflammation (46). There is also a well-established role of cholesterol and its oxidation products in atherosclerosis development. High cholesterol intake is associated with higher circulating quantities of low-density lipoprotein (LDL), as opposed to high-density lipoprotein (HDL) which promotes cellular efflux of cholesterol (47). Elevated levels of LDL lead to deposition in the arterial wall, where it is oxidized and aggregated, both of which further trigger immune activity. Modified LDL also activates TLRs on macrophages, triggering TLR signaling to mount an immune response, and are engulfed by macrophages to further amplify the immune response and trigger downstream cytokine production and inflammasome activation (48). Alongside the known mechanism in atherosclerosis development, the formation of aggregated cholesterol crystals and resulting NLRP3 inflammasome signaling has been implicated in the development of colon cancer (49). Both cholesterol and processed saturated fatty acids have been shown to modify the gut microbiota with effects on TLR4 (50); the resulting oxidative stress has also been shown to promote oxidized modifications of LDL.

Abundant evidence in animals and limited evidence in humans also suggests chemical additives in highly processed food, such as emulsifiers and sweeteners, directly contribute to a pro-inflammatory state (51). Results from an *in-vivo* study showed exposure to saccharin, a commonly used artificial sweetener, induced inflammation by elevating expression of proinflammatory cytokines, TNF-α and iNOS, and inducing changes to the microbiota (52). Another *in-*vivo study determined commonly used dietary emulsifiers, carboxymethylcellulose (CMC) and polysorbate-80 (P80), increased expression of genes pertaining to virulence of otherwise mutualistic bacteria and inflammation (53). Exposure to CMC and P80 has also been shown to increase intestinal permeability and result in changes in the gut microbiome that are associated with low-grade inflammation (54).

Furthermore, a study in a cohort of nearly six hundred people found a positive association between both emulsifier and highly processed food consumption and heightened inflammation and intestinal permeability; the association persisted after controlling for energy intake, BMI, and red and processed meat intake (55). The impact of CMC consumption on microbial composition has also been highlighted in humans using a randomized controlled-feeding study. Relative to control subjects, CMC consumption increased postprandial abdominal discomfort, reduced microbial diversity and led to changes in their metabolome, including a decrease in short-chain fatty acids (56). When looking at sweeteners, short-term and long-term results showed non-caloric artificial sweetener consumption induced alteration in the gut microbiota and glycemic response (57).

## Psychological determinants

### Chronic stress

Chronic stress is another proposed mechanism by which low income is associated with low-grade inflammation. Chronic stress has been linked with low-grade inflammation and chronic disease across several studies (58–61). Researchers have proposed that the activation of the HPA axis links chronic stress with low-grade inflammation. This phenomenon was supported across two viral-challenge studies, in which participants with recent exposure to a long-term threatening stressful experience displayed higher glucocorticoid resistance and increased production of inflammatory cytokines (62).

A randomized controlled trial in which participants were subjected to stress reduction interventions observed significant decreases in levels of CRP (63). Furthermore, in a study concerning childhood SES, investigators hypothesized early-life social adversity contributes to defensive biological programming which involves a heightened inflammatory state. The study measured CRP and IL-6 levels and conducted transcriptional profiling of healthy volunteers; the volunteers had no history of chronic disease but differed in spending their first 5 years of life in either low or high SES environments. The results suggested that participants raised in low SES environments had increased IL-6 production, which persisted after controlling for levels of perceived stress, smoking, adiposity, exercise, alcohol use, and sleep quality. Furthermore, transcriptional profiling revealed low SES was associated with an upregulation of genes involved in translating adrenergic signals to leukocyte transcription and genes with NF-κB response elements, which are characterized as proinflammatory genes. However, in the low SES cohort, there was also a downregulation of genes with response elements for the glucocorticoid receptor, which carry out the anti-inflammatory action of cortisol, commonly considered as the “stress hormone” (64). Both the inflammatory markers and transcriptional profiling results suggest a heightened inflammatory state among participants reared in low SES environments, which was hypothesized to act through resistance to cortisol output. While chronic stress has been consistently linked with low-grade inflammation, multifaceted analyses of social stressors, such as social strain and living in a single-parent family, and CRP have produced inconclusive results, suggesting that more research is necessary to characterize specific stressors that contribute to low-grade inflammation (65).

Chronic stress has also been proposed to contribute to low-grade inflammation through alterations to the microbiome. Abundant mouse and human studies have suggested a link between several types of stress and changes in the microbiome (66). In mice subjected to social disruption stress, levels of gut *Bacteroides* and *Parabacteroides* increased. Additionally, levels of *Coprococcus*, *Dorea*, and *Pseudobutyrivibrio* decreased, which were inversely correlated with IL-6 levels, and antibiotic administration blocked the stress-induced IL-6 increase (67). Similarly, a study assessing the effects of acute stress on pregnant women similarly observed stress-induced increases in IL-6, as well as TNF-α, were positively associated with abundance of *Bacteroides*. Increased IL-6 levels were also associated with abundance of *Prevotella* (68). Furthermore, in a cohort of Belgian children, high stress, as defined by negative life events and low parasympathetic activity, was associated with lower alpha diversity (69). Low alpha diversity has been linked to low-grade inflammation, with a potential mechanism being reduced access to complex carbohydrates and less production of short chain fatty acids (70). However, reverse causation is possible given that chronic stress may alter the habitat of the gut in ways that benefit survival of some microbes over others.

## Political determinants

The term “political determinants of health” was coined by Professor Daniel Dawes (71). The term is meant to contrast the more standard “social determinants of health” to communicate that many of the issues which fall under SDOH are not an innate part of a society. Rather, the cause and solution to these problems would require alterations in political decision making.

### Education access and quality

Educational attainment both captures early childhood determinants of health and largely predicts occupational and subsequently economic attainment (72). A gradient has emerged in the past 50 years in which higher levels of schooling are increasingly linked with better health outcomes and increased lifespan, partly through pathways influencing systemic inflammation. Individuals with lower educational levels may experience greater exposure to chronic psychosocial stress, reduced access to preventive healthcare, and higher rates of poor dietary and lifestyle factors. These factors are all associated with elevated inflammatory markers such as CRP and IL-6. The education-health gradient has been observed regardless of gender or race, although the health effects are stronger for women and white individuals (73). Here to, the association may be through reverse causation as greater health may better facilitate school performance.

### Childhood health

Educational context shapes childhood health outcomes, as most children spend up to 40 h of their week in school and receive approximately 35% of their daily nutrient intake while at school (74). As such, the school environment is a critical setting for interventions which reduce health inequities present in early childhood. Children with marginalized backgrounds commonly experience nutritional inequities that promote low-grade inflammation, including a lower consumption of fruits and vegetables and higher consumption of sodium, added sugars, and processed saturated fats (75, 76). Federally assisted meal programs such as the National School Lunch Program (NSLP) feed over 30 million children daily and have been considered essential in improving diet quality of underserved populations (75). NSLP lunches are required to meet nutritional standards laid out by the Dietary Guidelines for Americans (DGA). Children who participate in the NSLP have been shown to eat a healthier lunch than nonparticipants in the program regardless of income level or race (77). Even so, NSLP lunches allow for the selection of foods which meet DGA intake requirements for all major nutrients except for dietary fiber, which has been previously discussed to have strong implications for regulation of the immune system. Despite this requirement, students' average daily consumption of selected lunches did not meet intake recommendations for calcium, iron, fiber, and vitamins A and C (75). Nationally less than half of elementary students meet intake requirements for iron and vitamins A and C and very few consume the recommended amount of dietary fiber (78). However, the extent of these deficiencies is strongly shaped by the SDoH. Children from low-income households and under-resourced school districts are more likely to depend on school-provided meals as their primary source of nutrition and may have limited access to nutrient-rich foods outside of school. Socioeconomic status, neighborhood food environments, and school funding policies intersect to magnify nutritional inequities and their downstream inflammatory consequences. Increases in dietary fiber intake has been found to lower concentrations of serum CRP and fibrinogen in overweight and average weight adolescents, suggesting that dietary fiber may have protective effects against systemic inflammation (79, 80). Intake of vitamins A, C, and E have also been shown to have an inverse correlation with levels of CRP and IL-6 in children (81).

There is also evidence to suggest that higher education in a school environment which promotes attitudes of self-efficacy regarding personal health produces healthier behaviors throughout life (76). This relationship is potentially mediated by increased exposure to health education and interventions in schools, which are not offered equitably. Students at Title 1 schools, which are schools that serve a large percentage of low-income students, scored significantly lower when surveyed on nutritional knowledge and dietary behaviors compared to students of non-Title 1 schools. However, Title 1 students also demonstrated higher scores of self-efficacy when it came to selecting healthy meals which proved to be a more significant predictor of healthy dietary behaviors (82). School-based health centers (SBHC) provide another avenue for access to health services and the acquisition of healthy behaviors. Studies have shown that students with access to SBHCs showed greater participation in physical activity and met more nutritional intake guidelines compared to peers not using these facilities (83).

### Vocational differences

Over the past several decades, increasing globalization and automation in the workforce has increased the economic returns of higher education while reducing demand for less skilled labor. As income inequality has become more stratified by education level, correlations between education and mortality have become stronger (84). Higher education is also associated with increased likelihood of jobs providing non-wage related benefits, such as employer provided healthcare, paid leave, and retirement funds, all of which contribute to positive employment outcomes (85). Conversely, lower educational attainment coincides with increased occupational stress and precarity of employment conditions which encompass several inflammatory exposures (86).

Higher educational attainment has been inversely correlated with an individual's likelihood of participating in shift work (87). Shift work is broadly defined as a working schedule in which takes place outside of traditional daytime hours of 7 AM–6 PM (88). This type of work is commonly characterized by an irregular work schedule which includes regular evening and nighttime work, rotating shifts, or split shifts (89). Shift work has been identified as a risk factor for systemic inflammation which predisposes this class of employees to a host of other chronic disorders such as cardiovascular disease, cancer, and metabolic syndrome(88–91). A study of shift workers in Atlanta found a 93% higher concentration of CRP in collected blood samples compared to day workers. In addition, concentrations of the pro-inflammatory cytokines IL-1β and TNF-α were 96% and 20% higher among this cohort (92). Both IL-1β and TNF-α also have been shown to circulate in the body at higher levels following periods of sleep deprivation (93). In the same study, IL-6 concentrations in the blood were 190% higher among shift workers, while plasma cortisol levels were 39% higher (92).

The correlation between shift work and inflammation has been hypothesized to be related to the misalignment between workers' endogenous circadian rhythm and the sleep wake cycles shift work demands of employees (91). On average shift workers report sleeping 30–70 min less per day than dayworkers (90, 92). Furthermore, 10%–20% of shift workers experience shift work disorder (SWD), a condition characterized by insomnia or excessive daytime sleepiness in the context of a work schedule which interferes with one's endogenous sleep-wake cycle (90). A study of full-time shift workers found that conditions of short-term circadian misalignment consisting of a 12 hour inversion of the behavioral to environmental cycles resulted in an 11% increase to CRP produced within 24 h of misalignment (91).

### Occupational exposures

Employment precarity is informed by multiple factors such as low SES and minority status in a population (94, 95). Research has shown that decreasing education level is associated with increasing occupational precariousness. As the quality and stability of available employment declines, the risk that employees encounter occupational hazards increases (95). Lower educational attainment has been most strongly associated with heightened risk of exposure to chemical hazards and, to a lesser extent, physical and ergonomic hazards (95).

Lead exposure remains a common hazard of industrial jobs such as construction, often via inhalation of fumes or dust contaminated by the heavy metal. Although the American Conference of Governmental Industrial Hygienists (ACGIH) states that individuals may experience blood lead levels of 20 µg/dl without adverse effect, chronic exposure to lower levels of lead is associated with dysfunction to multiple organ systems (96). Chronic lead exposure has been correlated with significant increases to serum levels of TNF-α, IL-1β, and IL-6. Furthermore, levels of these pro-inflammatory cytokines are positively correlated with levels of angiogenic factors in lead exposed individuals, suggesting this inflammatory response may also promote cancer progression (97).

Ergonomic occupational hazards refer to working conditions which can cause strain on the body through repetitive motion, high exertion activities, or awkward posture (98). Notably, although moderate leisure time physical activity correlates with benefits to physical health and anti-inflammatory effects, occupational physical activity does not confer the same benefits. This phenomenon is referred to as the physical activity paradox (99, 100). Studies show occupational physical activity has a positive association with levels of CRP, suggesting this type of physical exertion has inflammatory effects (99, 100). Higher intensity of occupational physical activity was also associated with lower income levels (100).

## Neighborhood and built environment

There is a wealth of evidence that shows the neighborhoods and homes in which people live are strong determinants of health outcomes (101). Studies have displayed associations between low-grade inflammation and factors ranging from the physical qualities of the home itself (102) to broader neighborhood exposures like air pollution (103). The hypothesized mechanisms behind these associations range from microbial dysbiosis to activation of classic inflammatory pathways.

### Chemical exposures

Marginalized and low-income communities are disproportionately exposed to several classes of toxic chemicals, which have well-established harmful effects through widely encompassing sources of exposure(104–106). Inhalable particulate matter with a diameter of <2.5 μm (PM2.5), disproportionately affect marginalized communities, as higher concentrations of air pollution containing PM2.5 positively correlates with the percentage of Black residents, historical redlining score, and low-income census tracts(107–110) resulting in greater overall mortality (109, 110). Indoor air pollution is an especially significant risk factor, which sources of exposure including improper temperature control, poor indoor ventilation, smoking, and gas stove use (111, 112). Birth cohorts have displayed longitudinal associations between ambient PM2.5, PM10, and NO_2_ with the inflammation-association proteins IFN- γ and IL-12B (113). In experimental settings, PM2.5 exposure resulted in increased macrophage-mediated IFN-γ, IL-17, and IL-21 expression by T cells, as well as the formation of reactive oxygen species and secretion of IL-1β and TNF-α by macrophages (114, 115).

Additionally, heavy metal exposure, such as through contaminated water or food sources, disproportionately effects low-income and non-white communities (116–118). Heavy metals including lead, arsenic, and cadmium have been linked with chronic disease and immune dysregulation in both human and experimental settings (119). High ambient exposure to arsenic, chromium, cadmium, and nickel has been linked with increased rates of breast and colon cancers in marginalized communities (120). In animal models, lead exposure resulted in gut dysbiosis and an increase in opportunistic pathogens, as well as altered metabolism of key microbial species (121). Similarly, human studies showed that, depending on the route of exposure, lead exposure was associated with enhanced inflammatory responses including several proinflammatory cytokines and increased NF-κB signaling (122). Cadmium exposure has also been shown to result in lower abundance of SCFA-producing bacteria and increased TNF-α expression (123). Similarly, arsenic exposure increased proinflammatory cytokine expression, production of reactive oxygen species, and NF-κB signaling in experimental models (124–126).

There are also significant neighborhood and community-based disparities in exposure to persistent organic pollutants (POPs), which have been shown to promote inflammation. Housing conditions such as peeling paint, water leaks, cigarette smoke, pesticide residues, and old furniture expose residents to harmful chemicals and disproportionately affect urban communities with low SES (127). Exposures to POPs, including polybrominated diphenyl ethers (PBDEs), organochlorine pesticides (OCPs), polycyclic aromatic hydrocarbons (PAHs), and polychlorinated biphenyls (PCBs), are significantly higher in social housing multi-family units than in single family dwellings (128), as well as in old homes (129, 130), both of which are correlated with race and low income (131). Several epidemiologic studies have shown significant positive associations between serum concentrations of OCP and inflammatory biomarkers, including CRP, TNF-α, and IFN-γ . In *in-vivo* experimental studies, exposure to OCPs and PCBs through several routes increased expression of proinflammatory cytokines including IL-6, IL-10, TNF-α, and MCP-1 (132). Exposure to PCBs was also shown to promote leukocyte infiltration, activate NF-κB through the NEMO pathway, and alter metabolic processes of gut microbiota, resulting in decreases of SCFA-producing species(133–135). Likewise, exposure to PBDEs in experimental settings led to activation of NF-κB, NLRP3 inflammasome activation, and the production of reactive oxygen species due to mitochondrial dysfunction (136). Furthermore, high exposure to PAH's was significantly correlated with CRP and biomarkers of oxidative stress, as well as increased proinflammatory cytokine expression, in cohorts of pregnant women (137, 138). Benzo(α)pyrene, one of the most common PAH, induced oxidative stress, inflammatory cytokine expression, and NF-κB activation upon exposure to human endothelial cells and keratinocytes (139, 140). Given their persistence in the environment, the inflammatory effects of persistent organic pollutants span over long periods of time.

Alongside previously discussed exposures, phthalates, which are known endocrine disrupting chemicals, are additional sources of pro-inflammatory chemical exposures. There are well-established racial disparities in phthalate exposure (141–143). Weathering of construction materials in low-income housing (104, 144, 145), certain personal care products (146), and discount retailers (147), all of which have a greater presence in low-income and marginalized communities, are highly associated with high phthalate exposure (105). Several studies have suggested a causal link between phthalates and inflammation, showing phthalate exposure increased production of TNF-α by monocytes and macrophages, which has been observed in both *in-vitro* and *in-vivo* studies (148). Phthalate exposure has also been linked with heightened inflammation and oxidative stress in pregnant women (149).

### Housing conditions

In addition to abundant harmful chemical exposures, marginalized communities are disproportionately exposed to other inflammatory triggers due to poor housing quality (150). The quality of one's home physical environment has been linked with low-grade inflammation through exposures such as mold and poor temperature control. Moisture damage, for example, has been linked with increased CRP levels, as well as increased expression of IL-1β, IL-6, and TNF-α (151). A pilot study further observed an increase in innate immune activity, specifically pattern recognition receptor expression and cytokine release, among subjects exposed to moisture damage, with similar findings from *in-vitro* stimulation with model fungal substances (152). Exposure to isolated components of fungal specimens and mold from damp building environments also increased leukocyte infiltration in the bronchial space and gene expression of TNF-α in alveolar cell lines (153). Furthermore, an interventional study showed that repairing moisture damage decreased expression of IL-6, IL-4, and TNF-α (154).

Evidence suggests that improper temperature control and indoor ventilation, which are also associated with low-income housing, have implications for low-grade inflammation (155, 156). Short term temperature effects have been observed to correlate with IL-6 expression, plasminogen activator inhibitor-1 (PAI-1) levels, and CRP levels (157). Improper indoor temperature control has also resulted in increased airway inflammation, with increased expression of IgE and IgG, leukocytes, and inflammatory cytokines in *in-vivo* models (158), as well Th2 responses in asthmatic mice (159). Furthermore, in a randomized crossover trial, residence in homes with poor air conditioning in hot environments lead to increased intestinal fatty-acid binding protein (I-FABP), indicating decreased barrier integrity (160).

Overcrowded housing is another factor thought to contribute to chronic stress and related health outcomes in low-income communities (161). A population-based study observed early life household overcrowding, determined by number of people per room, to be associated with several markers of inflammation, including CRP and ICAM (162). In *in-vivo* models, mice subjected to high density housing conditions displayed increases in colonic CXCL1, TNF-α and IL22, hyperglycemia, and low-grade gut inflammation, alongside increases in corticosterone levels (163).

### Neighborhood conditions

Alongside material home characteristics, poor neighborhood conditions have also been implicated in low-grade inflammation. For instance, noise pollution is a significant environmental threat which disproportionately affects low-income and minoritized communities (164). An *in-vivo* study observed chronic noise exposure led to increased intestinal inflammation in rats with persistent elevation of TNF-α and IL1β, as well as alternation of the gut microbiome (165). Additional studies have observed an increase in IL-6 and other proinflammatory monocytes in response to noise, which has been hypothesized to occur in response to increases in stress hormone release upon noise exposure (166).

Longitudinal studies suggest the aspects of the built environment which influence walking and exercise habits may also contribute to the association with inflammation (167). A study investigating the association between walking behavior and built environment suggested that leisure walking was associated with retail zone walkability whereas commuter walking was associated with the number of walkable social destinations and street connectivity (167). Additional studies have observed correlations between gross population density, intersection density, and walkability indexes with physical activity (168). A cross-sectional survey of individual health survey responses also reported greater walking behavior in neighborhoods with more green space (169). Walking and exercise behaviors are key regulators of inflammation. Regular exercise has been shown to promote PGC1α, which has been shown to increase detoxification of ROS, promote vascularization, and suppress the production of inflammatory cytokines, including TNF-a and IL-6, in multiple *in-vitro* settings (170). Additionally, in a randomized controlled trial, increasing the steps per day reduced IL-6 levels, even after adjusting for obesity (171). A recent meta-analysis also reported lifelong exercise was associated with reduced levels of CRP and IL-6 (172).

Alongside the suggested influence on walking behavior, there are several reported health outcomes influenced by green space. Using multiple metrics of green space availability, including park cover, Normalized Different Vegetation Index (NDVI), and NatureScore, green space is positively associated with SES and percentages of non-Hispanic white residents (173). The presence of green space has also been linked with regulation of low-grade inflammation. Multiple metrics of greenness have been associated with lower CRP and IL-6, as well as white blood cell counts, B-cells and monocytes (174). In a cross-sectional study, residential greenness was also inversely associated with isoprostanes, which are robust indicators of systemic oxidative stress. Participants who lived in greener areas also had lower levels of sympathetic activation, supporting the hypothesis that stress levels may partially mediate the effect of green space on disease outcomes (175).

### Neighborhood access

Beyond intrinsic factors such as the built environment, neighborhoods affect other factors that are relevant to inflammation regulation. For instance, several neighborhoods within the United States are considered as “food deserts” or “food swamps.” Food deserts are regions in which people live more than 1 mile from a supermarket and lack healthy food options, while food swamps describe regions that are more than 1 mile form a supermarket and have a greater proportion of proinflammatory food options than fresh food (176). Food desert severity has been suggested to mediate the relationship between income and inflammation (177). Although assessment of neighborhood access to green space or health food is subject to limitations, including the fact that people are not necessarily limited to nearby grocery stores and green spaces, new methods to assess urban access beyond proximity measurements are emerging and should be incorporated in further inquiry (178).

Alongside neighborhoods with predominantly low-income residents and marginalized communities having reduced access to healthy food, there is greater availability of tobacco products (179). In marginalized communities tobacco products are more widely advertised, in both the frequency and nature of advertisement (180). Furthermore, a cross-sectional study of tobacco retailers in Washington, D.C. found predatory tobacco advertising tactics, specifically more appealing descriptors of tobacco, are more prevalent in census tracts with a greater proportion of Black residents. Similar findings were observed among Hispanic/Latino residents (181). Moreover, abundant evidence suggests flavored tobacco products are associated with increased initiation and prolonged use among youth and young adult tobacco users, compared to nonflavored products (182). Several studies have established a strong association between tobacco products and cigarette smoke with immune dysfunction, ranging from both inflammatory to suppressive effects (183). Namely, cigarettes contain known immunomodulatory toxins, including nicotine, carbon monoxide, acrolein, reactive oxidant substances, and more (183). In *in-vitro* models, cigarette smoke activates epithelial cells and induces chemokine expression but simultaneously impairs innate immune responses to pathogens by inhibiting secretion of key antimicrobial peptides (184). Results from several case-control studies also displayed increases in inflammatory markers including CRP, CCL17, and CCL11 (185).

## Social and community context

### Social support and cohesion

Social and community context broadly encompasses the intertwined relationship and community dynamics including social cohesion, discrimination, and relationships in the home, workplace, and community (186). Across diverse metrics of social support, perceived social support was inversely correlated with CRP, TNF-α, and IL-6 (187). While global measures of social support did not correlate with CRP or IL-6 in a different study, the frequency of positive social interactions associated with lower CRP in middle-aged adults (188). Perceived social cohesion, assessed by survey responses, has also been suggested to moderate the relationship between SES and CRP levels (189). While further studies are necessary to understand mechanisms by which this association may occur, studies hypothesize the influence of stress from poor cohesion or lack of social support, as well as the role of social support in encouraging other healthy behaviors, have implications for chronic inflammation. A cross-sectional study using results from the Healthy Aging in Neighborhoods of Diversity Across the Lifespan Study, a longitudinal study led by the National Institutes of Health, showed neighborhood social cohesion was associated with healthier behaviors, such as increased physical activity, less cigarette use, and healthier diets; social cohesion was more pronounced in white participants (190).

### Discrimination

The effects of discrimination based on race, ethnicity, migratory status, religion, class, and other factors on health outcomes have been increasingly studied, including in the context of inflammation (191). A longitudinal study assessing persistent exposure to various types of racial discrimination, including disrespectful treatment from co-workers, negative police encounters, or racial slurs, found that persistent exposure to discriminatory events was positively associated with a composite measure of inflammatory cytokines IL-1β, IL-2, IL-5, IL-6, IL-17, TNFα, and MIP-1b in a cohort of Black women (192). This association persisted after controlling for exposure to childhood adversity, BMI, and health behaviors including diet and exercise. Similar findings were concluded in a longitudinal study assessing the effects of self-report discrimination and community segregation on inflammatory cytokine expression in a cohort of 400 Black participants (193). The physiological processes hypothesized to link chronic social and economic disadvantage with racial and economic disparities have been describe as the “weathering hypothesis” (194, 195). Discrimination has been shown to over activate stress pathways, with investigators observing constructs of discrimination to be predictors and correlates of alterations in HPA axis activity (196). Several studies have also implicated the conserved transcriptional respond to adversity (CTRA), which describes increased transcription of pro-inflammatory immune response genes, as well as reduced expression of antiviral genes (197–200).

Exposure to discrimination and related stressors vary in nature and length of exposure, both of which have been suggested to influence the related effects on inflammation. Future inquiries should distinguish the effects of discrimination across various forms of chronic, low-grade inflammation. Discrimination and social disadvantage are also intertwined with previously described social conditions, such as occupational or air pollution exposures, which are associated with inflammation themselves. However, racial disparities across inflammatory markers and chronic disease are consistently observed even after controlling for several social conditions including educational attainment, household wealth, various health behaviors, usage of medication, and marriage (201–203).

### Exposure to violence

Among adolescents, home neighborhood murder rate and exposure to violence have been shown to interact to predict counts of classical monocytes (204). In a cohort of 1,391 adolescents followed up to 18 years of age, childhood exposure to violence was associated with elevated levels of soluble urokinase plasminogen activator receptor (suPAR) and IL-6 (205). Furthermore, a longitudinal study involving 236 children from the Chicago area concluded neighborhood violence was associated with increased signaling of NF-κB and activator protein 1 (AP-1) control pathways, as well as greater beta-adrenergic and lower glucocorticoid signaling (206).

### Adverse childhood experiences

Adverse childhood experiences (ACE), which are traumatic events that occur before the age of 18, have also been associated with higher inflammatory profiles. Specifically, among school-aged children, those who experienced parental substance abuse displayed higher levels of pro-inflammatory markers including IL-6 and IL-1β (207). The presence of ACE was also associated with an altered gut microbiota composition and response to cortisol in a cohort of pregnant women (68). Similarly, in a cohort of healthy adults, those with early childhood adversity demonstrated less response to cortisol, enrichment of inflammatory gene expression in stress responses, and increased activity of pro-inflammatory signaling overall in comparison to adults without trauma experience (208). Adolescents exposed to adversity also demonstrated elevated transcription of genes pertaining to myeloid lineage immune cells and CREB transcriptional activity, which has also been previously implicated in increased immune-related gene expression in the context of adverse experiences (209, 210).

## Healthcare access and quality

Healthcare access and quality describes the availability and accessibility of quality, timely, comprehensive, and respectful healthcare services and resources. Although limited research directly examines the association between this social determinant and inflammatory markers, particularly through mechanistic pathways, there are several notable findings to report.

### Delayed care

Insurance status, encompassed within healthcare access, has previously been associated with control of chronic conditions. Additionally, in a study utilizing NHANES participants from 1988–1994, those in the public/no insurance group had significantly elevated CRP compared to those with private insurance (211). Proposed mechanisms underlying this association include the observation that underinsured individuals often delay their care, resulting in worsening disease and inflammation for these individuals as they receive treatment only when their disease presents in a severe stage (212). A lack of awareness of disease may also contribute to chronic inflammation, as patients with lower insurance reimbursements also have significantly higher CRP/IL-6 levels post-surgery (213). In the case of a chronic inflammatory conditions such as lupus, those with public insurance have also been shown to have higher rates of hospitalization and readmissions compared to those on private insurance. Even among those who receive care, there is greater healthcare fragmentation, with patients often receiving services across multiple locations. Fragmented care is known to results in increased risk of comorbidities, hospitalizations, and overall healthcare costs (214). Together, these studies suggest that individuals without insurance are more likely to delay seeking care, which can lead to more advanced disease at presentation and a sustained inflammatory state that may remain undetected or unmanaged.

### Medication availability

Unsurprisingly, insurance status also affects an individual's ability to adhere to their medication regimen. A 2012 study found that patients hospitalized for cardiovascular disease were more likely to be incapable of staying adherent to their medications (215). A later study also showed that those on public insurance or uninsured had elevated CRP, which was associated with functional limitations (216). This association is clinically relevant, given that functional limitations have been shown to predict poor medication adherence in prior studies (217). This highlights a potential feedback loop where poor insurance coverage leads to delays in care and worse medication adherence, which leads to elevated inflammation and promotes functional impairments, which in turn makes it more difficult for patients to manage their chronic conditions.

## Discussion

**Figure 2:**
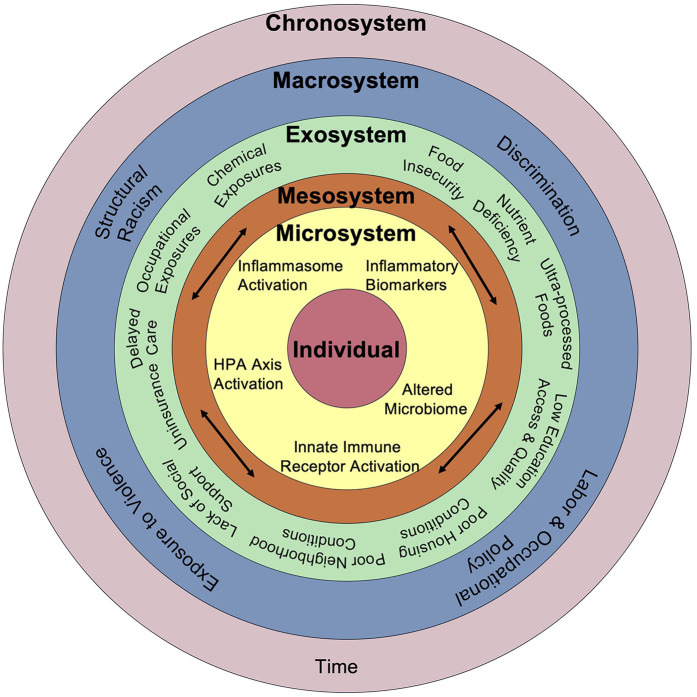
Comprehensive framework connecting social and biological drivers of chronic inflammation. Adapted from Bronfenbrenner's Social Ecological Model, this framework illustrates how social, environmental, and structural factors interact across nested levels—from individual biology to broader societal systems—to drive chronic inflammation through pathways including HPA axis activation, inflammasome signaling, altered microbiome composition, and innate immune receptor activation over time.

There are several mechanisms by which SDoH contribute to a heightened inflammatory state that elevates the risk of chronic disease development. Both an absence of resources and reduced frequency of lifestyle factors that mitigate inflammation, as well as toxic and stress-inducing exposures actively contribute to heightened inflammation. Placing these inflammatory triggers within the context of Bronfenbrenner's social-ecological model (218) identifies the exosystem as enriched for harmful exposures (Figure 2). However, in nearly every context where people are born, live, learn, work, play, and age, lifestyle factors and exposures influence regulation of the immune system. Furthermore, exposures beginning in childhood, and even *in utero*, can influence chronic low-grade inflammation later in life, as evidenced by fetal impacts of maternal deprivation, anti-inflammatory behaviors developed in school age, and the inflammatory impacts of adverse childhood experiences.

There are limitations of this review which should be further addressed in future inquiries. Firstly, chronic low-grade inflammation describes a broad condition. While measuring certain biomarkers, such as CRP and IL-6 is a common approach to assess this condition, levels of inflammatory cytokines, reactive oxygen species, and proxies for gut permeability have also been assessed to show evidence of systemic inflammation. The broad characterization of inflammation may also generalize more specific underlying processes. However, as there is strong evidence suggesting chronic low-grade inflammation, described broadly, underpins the development of several chronic diseases, this broader framework to conceptualize the large influence of SDoH on inflammatory disease risk may guide further inquiry into more specific pathways and mechanisms, as well as possible interventions to mitigate the burden of chronic disease.

Additionally, inflammatory conditions across social determinants of health often do not operate independently but are rather intertwined. For instance, the neighborhoods people live in can shape not only their built environment, but their opportunities for and access to economic prosperity, education, healthcare, and social cohesion. Researchers note deriving causal conclusions from observational studies is obscured by the interconnection between these factors, although experiments similarly present limitations in generalizability considering the cumulative effects of factors in real world settings (101).

Likewise, the directionality of reported observations is not fully clear from observational studies. For example, while lower educational attainment has been shown to be associated with greater CRP levels, further studies are necessary to confirm whether lack of education might contribute to elevated inflammation or greater inflammation might interfere with one's ability to attain higher education levels. Regardless of directionality, biomarkers of SDoH may still inform research, especially if the connections can be elucidated through multi-variant assessments of the SDoH against the specific biomarkers. For example, identifying specific biomarkers of the various SDoH parameters could reduce reliance on survey data and better determine which determinant is most impactful for each given individual. Ultimately, careful conclusions should be made from the presented research and when considering the application of these findings to interventions. For example, the noted biomarkers of inflammation present plausible mechanisms for the resultant harms to population health, however the solutions will require political and societal interventions rather than pharmacologic blockade of inflammatory pathways.

The abundant exposures that contribute to a proinflammatory state thought to underpin the development of several chronic diseases are largely connected to disparities in health outcomes. Several of the discussed mechanisms by which social contexts contribute to inflammation, from ultra-processed food to deteriorating housing conditions to discrimination predominantly affect low-income and historically marginalized communities. Through discriminatory practices such as historical redlining, Black and non-white communities were sequestered to neighborhoods which continue to be those with the highest inflammatory exposures (219). Persistent barriers across the five domains of SDoH for low-income and marginalized communities have been consistently shown to shape disparities in health outcomes (220), and the presented framework highlights inflammation as a key mechanism of this association.
